# A novel method for transforming *Geobacillus kaustophilus* with a chromosomal segment of *Bacillus subtilis* transferred via pLS20-dependent conjugation

**DOI:** 10.1186/s12934-022-01759-8

**Published:** 2022-03-08

**Authors:** Kotaro Mori, Kaho Fukui, Ryotaro Amatsu, Shu Ishikawa, Valeria Verrone, Anil Wipat, Wilfried J. J. Meijer, Ken-ichi Yoshida

**Affiliations:** 1 Department of Science, Technology and Innovation, Kobe University, 1-1 Rokkodai, Nada, Kobe, 657 5801 Japan; 2 School of Computing, Newcastle University, 1 Science Square, Science Central, Newcastle upon Tyne, NE4 5TG UK; 3 Centro de Biología Molecular ‘Severo Ochoa’, CSIC-UAM Universidad Autónoma Madrid, Canto Blanco, 28049 Madrid, Spain

**Keywords:** *Bacillus subtilis*, Conjugation, *Geobacillus kaustophilus*, Transformation

## Abstract

**Background:**

*Geobacillus kaustophilus* is a thermophilic Gram-positive bacterium. Methods for its transformation are still under development. Earlier studies have demonstrated that pLS20catΔoriT mobilized the resident mobile plasmids from *Bacillus subtilis* to *G. kaustophilus* and transferred long segments of chromosome from one cell to another between *B. subtilis*.

**Results:**

In this study, we applied mobilization of the *B. subtilis* chromosome mediated by pLS20catΔoriT to transform *G. kaustophilus*. We constructed a gene cassette to be integrated into *G. kaustophilus* and designed it within the *B. subtilis* chromosome. The pLS20catΔoriT-mediated conjugation successfully transferred the gene cassette from the *B. subtilis* chromosome into the *G. kaustophilus* allowing for the desired genetic transformation.

**Conclusions:**

This transformation approach described here will provide a new tool to facilitate the flexible genetic manipulation of *G. kaustophilus*.

**Supplementary Information:**

The online version contains supplementary material available at 10.1186/s12934-022-01759-8.

## Background

Bacteria proliferate by asexual cell division, and therefore their diversity in genetic traits is likely due to spontaneous mutation. However, bacteria can enhance their genetic diversity through unique mechanisms known as horizontal gene transfer [1]. Horizontal gene transfer takes three distinct mechanistic forms: transformation, transduction, and conjugation. Plasmids are extrachromosomal DNA molecules that play a crucial role in conjugation. Conjugative plasmids encode various elements required for conjugation, including enzymes such as relaxase, recruiting its partner proteins to form a nucleoprotein complex named relaxosome, various proteins composing translocation apparatus, and a DNA stretch with the *oriT* sequence where the conjugation-specific replication initiates. During conjugation, a donor cell attaches to a suitable recipient cell. The relaxase enzyme attaches to the 5´-end of the single-stranded DNA to form a strand-specific nick at the *oriT* site. It results in the formation of relaxosome within the donor cell. Then, the membrane-bound translocation apparatus synthesized on the surface of the donor cell recruits the relaxosome. The relaxase enzyme navigates the single-stranded DNA transferred through the translocation apparatus, and the transferred DNA is converted into double-stranded DNA in the recipient cell. On the other hand, non-conjugative plasmids do not encode any of the elements mentioned above and thus cannot transfer themselves [2]. However, when a non-conjugative plasmid has the *oriT* sequence, it becomes a mobilizable plasmid and can be transferred from one cell to another by the helper function of the conjugative plasmid [3].

pLS20 is a 65-kb conjugative plasmid originally isolated from *Bacillus subtilis* var. *natto*, that exhibits conjugative self-transfer characteristics among various *Bacillus* species, including *B. subtilis*, *Bacillus anthracis*, *Bacillus cereus*, *Bacillus licheniformis*, *Bacillus megaterium*, *Bacillus pumilus*, and *Bacillus thuringiensis* [4]. The conjugative transfer of pLS20 is achieved by mixing the donor and recipient cells in a liquid medium [5]. The genes responsible for conjugation in pLS20 (genes 28–74) are clustered in a large operon that is controlled by a strong promoter, named Pc. Regulation of the pLS20 conjugation genes has been studied in detail [6]. The gene 27 on pLS20 encodes the master regulator of the conjugation operon, Rco_pLS20_, that binds to the operator site within the Pc region to repress the expression of conjugation operon in the ground state. The product of gene 25 is Rap_pLS20_ (an anti-repressor that antagonizes Rco_pLS20_), which can relieve the repression caused by Rco_pLS20_ to induce the conjugation operon. On the other hand, the gene 26 synthesizes a small polypeptide that is secreted from the donor cells and processed to release its C-terminal part (five amino acid residues length) as the quorum sensing peptide Phr*_pLS20_, which then accumulates in the culture medium. Phr*_pLS20_ is taken back into cells when its accumulation reaches a certain level, inactivating the anti-repressor activity of Rap_pLS20_. Therefore, the expression of conjugation genes returns to the ground state as Rco_pLS20_ represses the Pc region to prevent further conjugation [7]. Ectopic overproduction of Rap_pLS20_ diminishes the effect of Phr*_pLS20_, and the expression levels of conjugation genes enhances, resulting in elevated conjugation efficiency [7].

*Geobacillus kaustophilus* HTA426 is a thermophilic Gram-positive bacterium isolated from deep-sea sediments collected from the Mariana Trench [8]. HTA426 grows at higher temperatures from 48 to 74 °C (optimally at 60 °C) and is considered a prospective chassis to construct a high-temperature resistant cell factory. This has the advantage of preventing contamination by mesophilic bacteria and lowering the cost of controlling the fermentation heat. The generation of HTA426 derivatives with improved industrial characteristics requires methods that allow genetic modification of its genome. Because the strain is reluctant to genetic modification by natural competence and electroporation, an approach was developed based on a Gram-negative conjugation system [9]. However, because this strategy relies on counterselection, it has the drawback of being unable to select for mutations that adversely affect the growth. Therefore, a better technique is warranted to manipulate the genome of *G. kaustophilus*.

In the present study, we developed a strategy that permits the transfer of a chromosomal segment of *B. subtilis* into *G. kaustophilus* and provides flexible chromosomal manipulation in *G. kaustophilus*. In a previous study, we harnessed the helper function of pLS20cat to transfer the a-la-carte-designed mobilizable plasmid pGK1 from an engineered donor strain of *B. subtilis* to a recipient strain of *G. kaustophilus*. However, the mobilization efficiency of pGK1 transfer was 50-fold lower than the mobilization of plasmid pGR16B [10] between *B. subtilis* [11]. Similarly, when the pLS20 *oriT* sequence was inserted into the chromosome, pLS20catΔoriT, a derivative of pLS20 lacking *oriT*, enabled the transfer of large chromosomal segments between *B. subtilis* cells, but the efficiency was also reduced significantly [5]. Here, we have studied several conditions and have applied the results to improve efficiencies to transfer chromosomal DNA segments from *B. subtilis* into *G. kaustophilus.*

## Results

### Additional conditions to enhance the efficiency of pLS20-mediated conjugation.

The pLS20-mediated conjugative transfer of the mobilizable plasmid pGR16B between *B. subtilis* donor and recipient cells was effective that 0.1% of the total recipient cells acquired pGR16B. Whereas plasmid pGK1 transfer from *B. subtilis* to *G. kaustophilus* was significantly less effective [11]. In addition, the pLS20-mediated chromosomal DNA transfer between *B. subtilis* was also reduced [5]. Therefore, a considerable decrease in efficiency is expected in the heterologous chromosomal DNA transfer from *B. subtilis* to *G. kaustophilus*, which is the aim of this study. In order to enhance the efficiency of DNA transfer from *B. subtilis* to *G. kaustophilus*, certain measures were employed: overexpressing *rap*_*pLS20*_ in donor cells to counteract repression of conjugation by quorum sensing and pre-methylating DNA by expressing the *dam* methylase gene in donor cells to prevent degradation of the transferred DNA by the restriction modification system present in *G. kaustophilus* [5, 11].

**Fig. 1:**
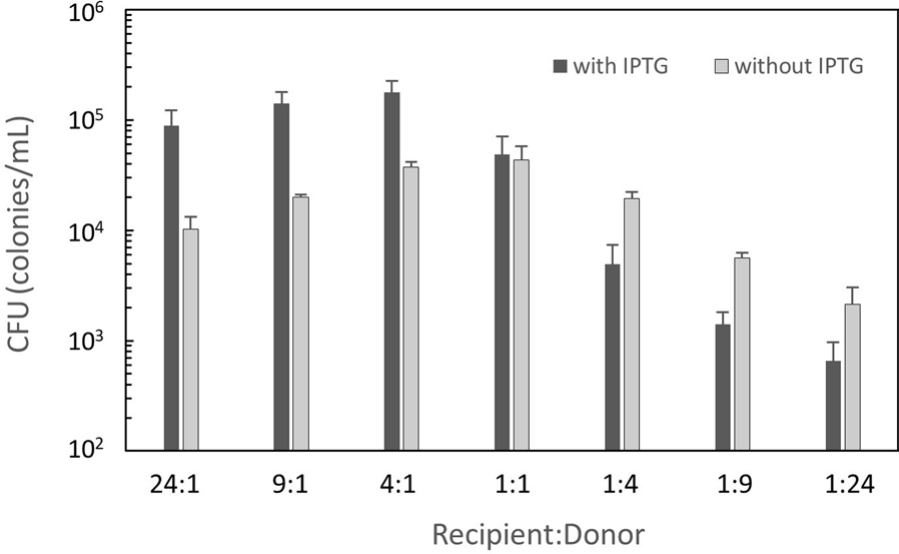
CFU of transconjugants. Donor strain KV9 and recipient strain KV7 were at various ratios for the conjugative transfer of plasmid pGR16B. KV9 was grown with and without IPTG before the conjugation. Colonies of transconjugants were counted to calculate CFU. The values are mean ± SD from three independent experiments

Besides these measures, we also wondered if the conjugation efficiency can be improved by using ratios of donor and recipient cells in mating mixtures different from the 1:1 ratio that has been standardly used in conjugation experiments. To study this, we determined the conjugative transfer of plasmid pGR16B between *B. subtilis,* we compared the colony formation unit (CFU) of transconjugants using different ratios of donor (strain KV9) and recipient (strain KV7) (Table 1) [12]. This setup allows to detect subtle differences because pGR16B mobilization is very efficient. In the absence of IPTG, *rap*_*pLS20*_ is only expressed from its native locus on the plasmid. As shown in Fig. 1, the highest CFU of transconjugants was observed at a recipient:donor ratio of 1:1. In the presence of IPTG, *rap*_*pLS20*_ is induced from the ectopic locus, which results in an increase of the conjugation efficiency. Under these conditions, the highest CFU of transconjugants was obtained at a recipient:donor ratio of 4:1, although the difference was not statistically significant [12]. These results suggest that the recipient:donor ratio of 4:1 might promote the conjugation efficiency for heterologous DNA transfer from *B. subtilis* to *G. kaustophilus*.

**Table 1:** Bacterial strains and plasmids used in this study

Strain	Genotype	Source or reference
*B. subtilis*		
168	*trpC2*	Laboratory stock
KV7	*trpC2* Δ*comK*::*tet*	[12]
KV9	*trpC, amyE*::(*Pspank-rappLS20 spc*) pGR16B pLS20catΔoriT	[12]
YNB104	*trpC2 epr*::(*PrpsO-dam ble*) pLS20catΔoriT pGK1 (*kan*)	[5]
YNB111	*trpC2 amyE*::(*Pspank-rap*_*pLS20*_* spc*) *yhfK*::(*oriT *_*pLS20*_* erm*) pLS20catΔoriT	[5]
YNB202	*trpC2 aprE*::(Δ*iolQ*_*GK*_::*kan*)	This study
YNB211	*trpC2 amyE*::(*Pspank-rap*_*pLS20*_* spc*) *epr*::(*PrpsO-dam ble*) *yhfK*::(*oriT*_*pLS20*_* erm*) pLS20catΔoriT	This study
YNB213	*trpC2 amyE*::(*Pspank-rap*_*pLS20*_* spc*) *epr*::(*PrpsO-dam ble*) *yhfK*::(*oriT*_*pLS20*_* erm*) pLS20catΔoriT *aprE*::(Δ*iolQ*_*GK*_*::kan*)	This study
*G. kaustophilus*		
MK244	Δ*pyrF* Δ*pyrR* Δ*GK1378–GK1390* Δ*GK0343–GK0346*	[13]
MR01	Δ*pyrF* Δ*pyrR* Δ*GK1378–GK1390* Δ*GK0343–GK0346* Δ*iolQ*_*GK*_::*kan*	This study
Plasmid		
pGR16B	*amp erm oriT*_*pLS20*_	[10]
pLS20catΔoriT	*cat* Δ*oriT*_*pLS20*_	[5]
pUCG18T	*amp kan*(TK101) *oripUC oripBST1 oriTInc-P1*	[9]

**Fig. 2:**
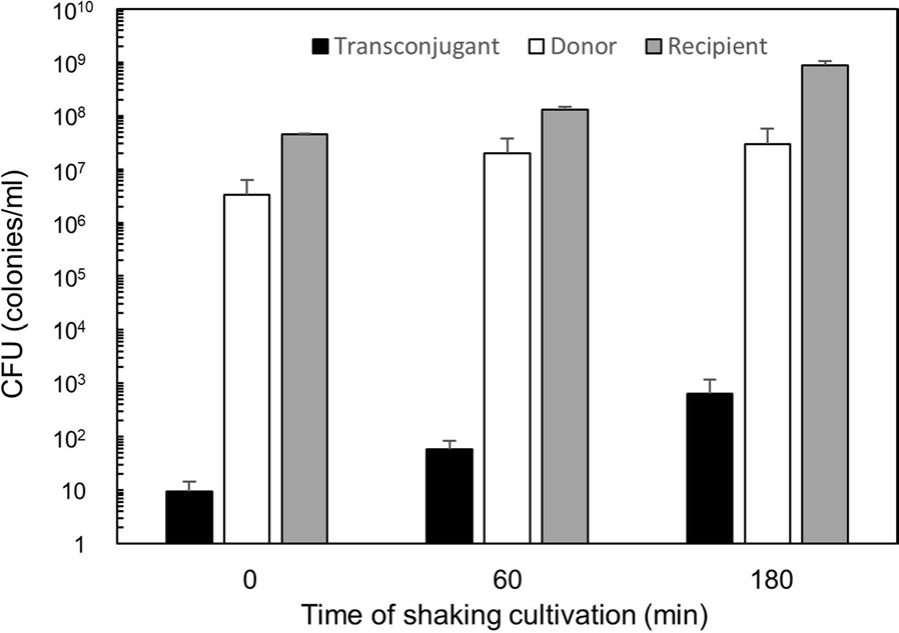
CFUs of transconjugants (solid), donors (open), and recipients (gray). Donor strain YNB213 and recipient strain KV7 were mixed at a 4:1 ratio of recipient: donor for the conjugative transfer of chromosomal segment containing the Δ*iolQ*_*GK*_::*kan* cassette. YNB213 was grown in the presence of IPTG before the conjugation. After keeping the mixed donor and recipient for conjugation, the mixture was allowed to grow with shaking for the time indicated. Colonies of transconjugants, donors, and recipients were counted to calculate CFU. The values are mean ± SD from three independent experiments

It is likely that chromosomal DNA segments that are transferred via conjugation require time for recombination to integrate into the chromosome of the recipient cell and to express the genes, we investigated the effect of shaking cultivation after the stationary mating to allow the transconjugant to adopt the newly acquired genes. For conjugation recipient strain KV7 and donor strain YNB213 of *B. subtilis* were combined in a 4:1 ratio to stand for 90 min and then subjected to shaking cultivation for the cells to grow. Figure 2 shows that the CFU of transconjugant incremented at increasing the shaking periods, reaching a maximum of 68-fold using incubation period of 180 min. The incubation period also allows growth of the donor and recipient cells. However, whereas the number of transconjugants increased 68-fold, the numbers of donor and recipient cells only increased 9- and 19-fold, respectively. After the 180 min of shaking cultivation, the total CFU of all the donor, recipient, and transconjugant became very high up to 10^9^, suggesting that the culture might almost have reached saturation.

### Transferring *B. subtilis* chromosomal DNA to *G. kaustophilus*.

The conjugative transfer of chromosomal DNA mediated by pLS20 requires *oriT*_*pLS20*_ integrated into the donor cell chromosome. Moreover, efficiency varies depending on the distance between *oriT*_*pLS20*_ and the region where the selection marker was placed; the maximum efficiency was observed when *oriT*_*pLS20*_ and the marker were 9.5 kb apart [5]. In our study, we placed the *oriT*_*pLS20*_ at *yhfK* locus and the Δ*iolQ*_*GK*_::*kan* cassette at *aprE* locus on the chromosome of *B. subtilis*, 9.5 kb apart from each other. The donor strain YNB213 (Table 1) was constructed by introducing the following three elements: (i) IPTG-inducible *rap*_*pLS20*_, which elevates conjugation efficiency [11]; (ii) *dam*, which methylates DNA to be protected from the restriction/modification system in *G. kaustophilus* [9]; and (iii) the *oriT*_*pLS20*_-less helper plasmid, pLS20catΔoriT [11]. *G. kaustophilus* strain MK244 was used as the recipient strain [13]. MK244 and YNB213 were mixed in a 4:1 recipient: donor ratio and stand at 37 °C for 90 min, which was previously reported as the duration required for chromosomal transfer in *B. subtilis* [5]. The mixture was then cultured at 60 °C for 180 min with shaking, after which the cells were spread on agar plates containing kanamycin followed by incubation at 60 °C. The efficiency of transconjugants in forming colonies was 1.40 ± 1.21 × 10^−10^ (transconjugant/recipient). One of the transconjugants was isolated and designated *G. kaustophilus* strain MR01.

### Correct transformation of the transconjugant.

**Fig. 3:**
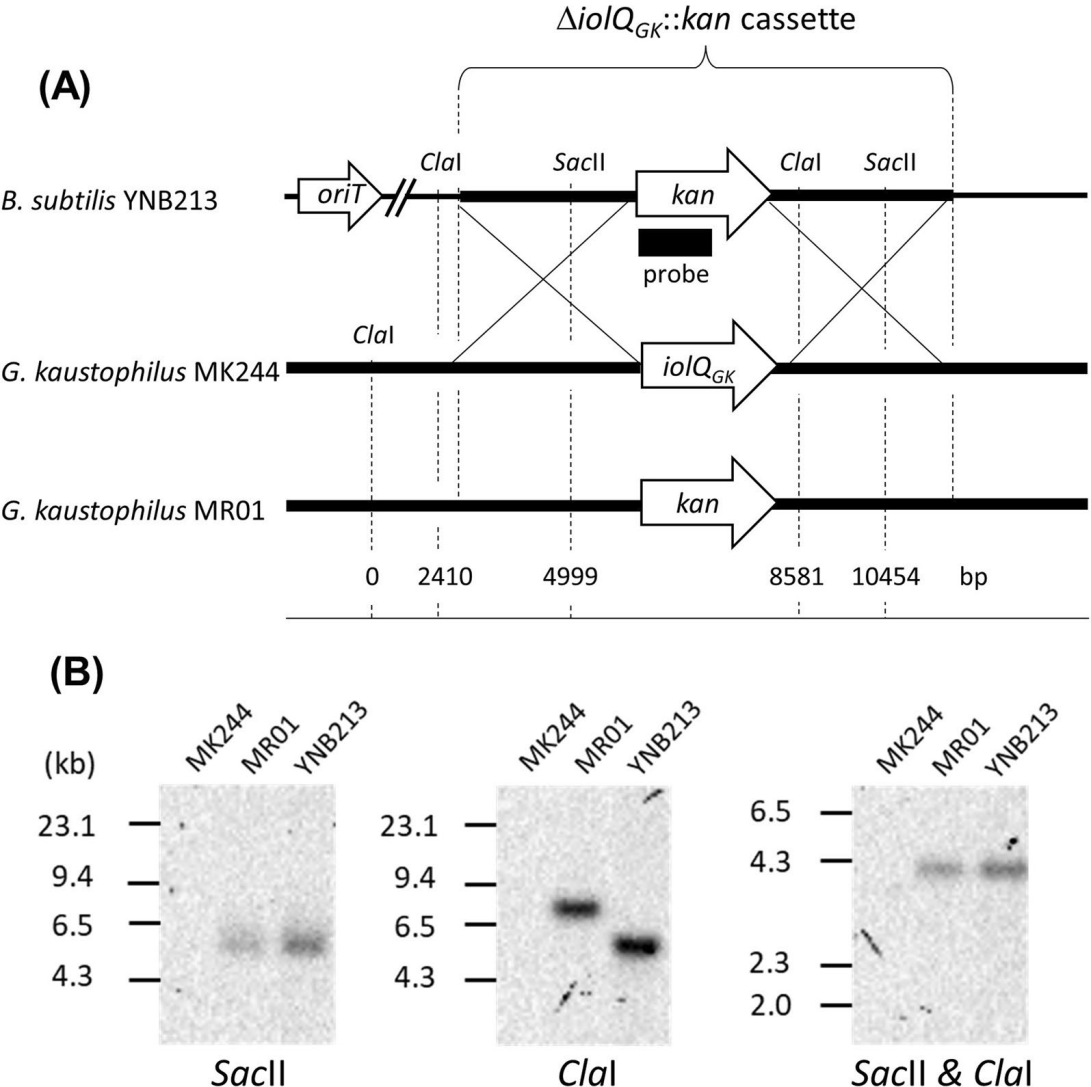
Southern blot analysis of strains of *B. subtilis* and *G. kaustophilus.* **A** Schematic organization of the relevant chromosomal loci of *B. subtilis* YNB213 and *G. kaustophilus* MK244 and MR01. Chromosomal segments corresponding to *B. subtilis* and *G. kaustophilus* are shown as thinner and thicker lines, respectively. The Δ*iolQ*_*GK*_::*kan* cassette set in the chromosome of YNB213 is shown at the top in brackets. *Cla*I and *Sac*II sites are indicated with a scale underneath representing the relative distance (bp) from the most upstream *Cla*I site. The *kan* and *iolQ*_*GK*_ genes and *oriT* are shown as arrowheads. The stretch of probe set within *kan* for the Southern blot analysis is indicated as a solid rectangle. **B** Results of the Southern blot analysis using the RNA probe to detect *kan*. Chromosomal DNAs of MK244, YNB213, and MR01 were digested by *Cla*I and/or *Sac*II as indicated and subjected to the analysis. No signal was detected in MK244, since it does not have *kan*. In YNB213 and MR01, a single band specific to each was detected. Only for the digestion by *Cla*I, the length of the DNA fragments containing *kan* varies due to the difference in the restriction sites. The experiments were repeated at least three times with similar results, and the representative data were shown

Southern blotting was used to analyze the genomic DNA of *G. kaustophilus* MR01 to ensure that the clone was not the result of a non-specific recombination event (Fig. 3). In this experiment, we used two restriction enzymes, *Sac*II and *Cla*I, to digest the chromosomes in three combinations: *Sac*II alone, *Cla*I alone, and both *Sac*II and *Cla*I. The length of the fragments obtained from each cleavage site is shown in the figure below (Fig. 3). The probe corresponded to part of the coding region of kanamycin (*kan)*. Considering that the chromosomal DNA to be analyzed was completely digested, the number of bands detected is proportional to the number of *kan* on the chromosome, i.e., the presence of two or more bands indicates non-specific introduction into the chromosome. In contrast, the presence of one band indicates a specific introduction to a particular locus. These results indicate that only the respective target with the predicted length was detected in the lanes of MR01 and YNB213 cleaved with the two restriction enzymes (Fig. 3). According to this novel method, an effective construction of MR01 was confirmed in terms of its genome structure.

**Fig. 4:**
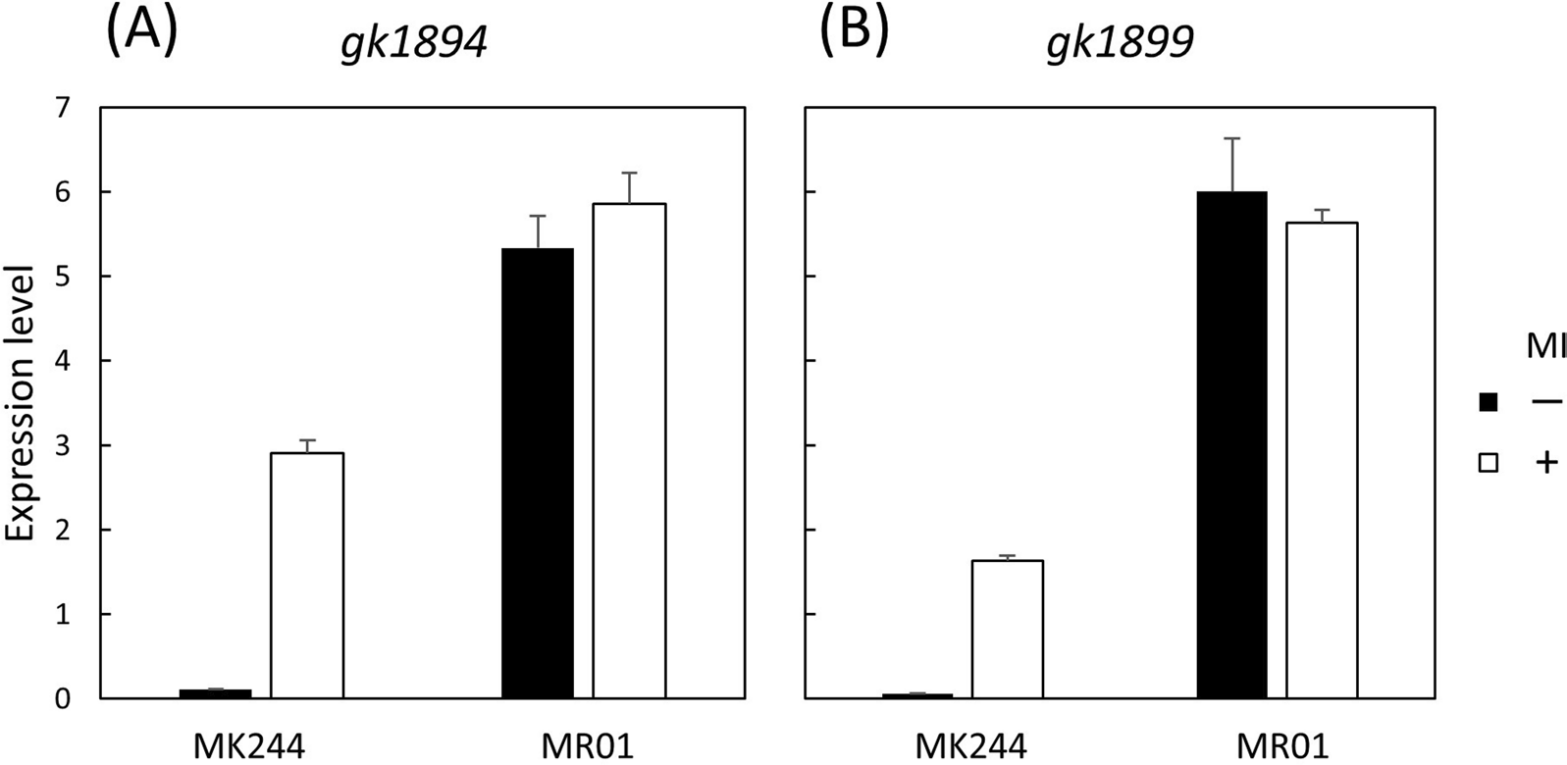
Quantitative RT-PCR analysis of the *iol* transcripts. Strains MK244 and MR01 were grown with (MI +) and without (MI –) 10 mM *myo*-inositol to extract the total RNA, which was subjected to quantitative RT-PCR to quantify the transcripts of the two tandem *iol* operons. The latter and former operons contain *gk1894* (**A**) and *gk1899* (**B**) as the first open reading frames, respectively, transcripts of which were quantified and normalized using the comparative C_t_ method with the internal control of *gk0103* transcript. The values are mean ± SD from three independent experiments

The *iolQ*_*GK*_ gene, which was deleted from the MR01 genome, encodes the repressor for transcriptional regulation of the two *iol* operons involved in inositol catabolism in *G. kaustophilus* [14, 15]. Even in the absence of *myo*-inositol (inducer) in the culture medium, both *iol* operons in MR01 were found to be transcribed constitutively (Fig. 4). These results corroborated our previous findings with another *iolQ*_*GK*_ mutant [14], indicating that the conjugation/transformation was successful, as evidenced by this phenotypic change.

## Discussion

The present study demonstrates the use of conjugative plasmid pLS20 to transfer chromosomal DNA from *B. subtilis* to *G. kaustophilus* as a genetic manipulation method. This new approach differs from the conventional gene manipulation technique for *G. kaustophilus,* which involves the two-step processes: the first step is to create the DNA segment to be delivered to *G. kaustophilus* on the *B. subtilis* chromosome, and the second step is the conjugative transfer to insert the DNA into the *G. kaustophilus* chromosome. Furthermore, it can solve the problems and difficulties of conventional techniques using Gram-negative conjugation that require the counterselection marker [16]. However, improvement is required for this approach, particularly the low conjugation/transformation efficiency, which is the most severe problem. Transconjugants are currently obtained by mating donors and recipients in a large-scale liquid culture and then concentrating them by centrifugation followed by spreading on agar plates. It is necessary to reduce the scale of this culture by improving efficiency.

The following is a list of possible future attempts to increase conjugation/transformation efficiency. The first is to promote the survival rate of recipient *G. kaustophilus* on agar medium. Previous studies have shown that conjugation can compromise the integrity of the cell wall resulting in the death of the recipient cells [17]. A modified lysogeny broth (LB) was shown to be suitable for accelerating the growth rate and reverting *G. kaustophilus* protoplasts into normal growing cells; the modified LB was supplemented with 10% lactose and 10 mM MgCl_2_ to serve as a regeneration medium [18]. Since the osmotic pressure of the medium is adjusted by lactose, it is hypothesized that the osmotic stress induced by probable cell wall damage during conjugation might be alleviated, thereby increasing the survival rate could be increased. The second point is to control the induction of *rap*_*pLS20*_ more effectively. The enhanced expression of genes required for conjugation is induced by the addition of IPTG to *rap*_*pLS20*_. However, we previously observed that inducing *rap*_*pLS20*_ in donors resulted in cell death [12]. The overexpression of the conjugation apparatus might cause a significant burden on the donor cell. Considering that the donor cell has only one copy of the mobilizable region of the genome—transmitted by the conjugation—the current conditions for inducing *rap*_*pLS20*_ may be too extreme and should be optimized in future studies. The final point is to adjust the temperature and timing for the mating between *B. subtilis* donor and *G. kaustophilus* recipient. In our study, mating was accomplished by mixing the liquid cultures containing donor and recipient cells and incubating the mixture at 37 °C for 90 min without shaking, as reported to be required for chromosome transmission between *B. subtilis* donor and recipient [5]. However, in the present study, *G. kaustophilus* is the recipient bacterium, which requires a higher growth temperature, and incubation at 37 °C for 90 min might be inadequate. Higher temperature, on the other hand, could be harmful for *B. subtilis* donor but it may survive up to 48 °C. Therefore, optimizing the temperature and timing for mating *B. subtilis* donor and *G. kaustophilus* recipient may help to improve the conjugation efficiency.

The novel finding described here suggests that conjugation/transformation via the chromosomal DNA transfer from *B. subtilis* can be further developed as a next generation tool for genetic engineering and can be applied to other bacteria, besides *G. kaustophilus* that have previously been proven difficult to alter genetically. This transformation method based on the conjugative transfer of chromosomal DNA is advanced in that the final target gene configuration can be freely designed and prepared on the *B. subtilis* chromosome, and then incorporated into the chromosome of the target microorganism. This is due to the remarkable plasticity of the *B. subtilis* chromosome, which validates the uniqueness of this method. For thermophiles, the disadvantage is that the thermostable antibiotic resistance gene available at present is limited to *kan* as used in this study, and it is desirable to develop additional thermostable markers to accomplish multiple gene transfer.

## Conclusions

Herein, we present a unique approach for transferring chromosomal DNA from *B. subtilis* to transform *G. kaustophilus,* which is mediated by conjugative plasmid pLS20. The method outlined here will be beneficial as a tool for flexible genetic manipulation of *G. kaustophilus*.

## Methods

### Bacterial strains, plasmids, and primers

The strains and plasmids used in this study are listed in Table 1. The primers used are shown in Additional file 1. The strains were cultured in LB medium (Becton, Dickinson and Company, Franklin Lakes, NJ). Antibiotics were added to the culture as needed at the following concentrations: 7.5 mg/L kanamycin, 5 mg/mL chloramphenicol, 1 mg/L erythromycin, 100 mg/L spectinomycin, 12.5 mg/L tetracycline, and 8 mg/L phleomycin.

### Preparation of strains

To construct the *B. subtilis* donor strains, a Δ*iolQ*_*GK*_::*kan* cassette was constructed by ligating five PCR-fragments and inserting it into the *aprE* locus of *B. subtilis* chromosome as described below. The fragments 1 and 5 corresponded to the upstream and downstream regions of the *aprE* locus of *B. subtilis*, which were amplified by PCR using primer sets aprE-U-f1/aprE-U-r and aprE-D-f2/aprE-D-r (Additional file 1), respectively, using chromosomal DNA strain 168 as a template. The fragments 2 and 4 were the upstream and downstream regions of *iolQ*_*GK*_, amplified using the primer sets degA-U-f/degA-U-r2 and degA-D-f2/degA-D-r (Additional file 1), respectively, from *G. kaustophilus* MK244 chromosome as a template. Finally, fragment 3 corresponds to the thermostable version of kanamycin resistance gene (*kan*) functioning at high temperatures (60 °C) to grow *G. kaustophilus*, which was amplified using the primer set kan-f/kan-r2 (Additional file 1) from pUCG18T [9]. All the fragments were linked from 1 to 5 in this order by recombinant PCR using the primer pair aprE-U-f3-nested/ aprE-D-r2-nested (Additional file 1) to form the complete gene cassette.

As described previously, the gene cassette was introduced into *B. subtilis* strain 168 to generate strain YNB202 that is kanamycin-resistant by natural competence [19] (Table 1). Strain YNB211 [*epr*::(P*rpsO-dam ble*)] was made to be phleomycin-resistant transforming strain YNB111 with DNA of YNB104 (Table 1). Finally, strain YNB213 [*aprE*::(Δ*iolQ*_*GK*_::*kan*)] was obtained as kanamycin-resistant by transforming YNB211 with DNA of YNB202 (Table 1).

### Conjugation

Conjugation was performed based on the method described previously [5]. Each of the bacterial strains used as the donor and the recipients was grown until the cells grew to OD_600_ 0.5 in LB liquid medium with shaking at 200 rpm at appropriate temperatures: for *B. subtilis* and *G. kaustophilus*, 37 °C and 60 °C, respectively. The donor and recipient cultures were mixed in various ratios as indicated and incubated without shaking at 37 °C for 15 and 90 min to transfer plasmid and chromosomal DNA, respectively. When necessary, the mixture was incubated further at 37 °C and 60 °C for *B. subtilis* and *G. kaustophilus*, respectively, up to 180 min with shaking at 200 rpm. CFUs of the donor, recipient, and transconjugant were determined by spreading the conjugation mixture on appropriate selection plates incubated at 37 °C and 60 °C for *B. subtilis* and *G. kaustophilus*, respectively. When needed, the conjugation mixture was diluted appropriately to count the colonies or concentrated by centrifugation before spreading on the plates when the conjugation was less efficient.

### Southern blotting

The probe used was prepared as follows. The DNA of pUCG18T was used as the template to amplify part of the kanamycin resistance gene by PCR using the primer set kan-probe-f/kan-probe-r (Additional file 1). To obtain the DIG-labeled RNA probe, the PCR fragment was subjected to in- vitro transcription using T7 RNA Polymerase (Roche, Basel, Switzerland). Genomic DNAs of bacteria were digested by *Cla*I and *Sac*II, electrophoresed in an agarose gel, treated with 0.25 M HCl for 30 min and then with 1.5 M NaCl/0.5 M NaOH for 30 min, and transferred to a positively charged nylon membrane Amersham Hybond-N + (GE Healthcare, Chicago, IL) using Vacuum Transfer BS-31 (Bio Craft, Tokyo, Japan). The transferred DNAs were crosslinked to the membrane with UV crosslinker CL-1000 (UVP, Upland, CA). Hybridization was performed using DIG Easy Hyb (Roche, Basel, Switzerland) according to the standard protocol. The membrane was blocked with Blocking Reagent (Roche). Anti-DIG-AP Fab fragment (Roche) was diluted 10,000 times in Blocking Reagent and incubated with the membrane at room temperature for 30 min. After the incubation, the membrane was washed thoroughly and incubated in detection buffer [0.1 M Tris, 0.1 M NaCl, 50 mM MgCl_2_ (pH 9.5)] containing the chemiluminescent substrate, disodium 3-(4-methoxyspiro {1,2-dioxetane-3,2'-(5'-chloro) tricyclo [3.3.1.1^3,7^] decan}-4-yl) phenyl phosphate (CSPD, Roche), to detect the signal using ChemiDoc XRS + (Bio-Rad, Hercules, CA) according to the standard protocol.

### Analysis of *iol* transcripts

Levels of transcription of the two *iol* operons of *G. kaustophilus* [14, 15] were quantified using quantitative RT-PCR. Strains MK244 and MR01 were grown aerobically in a minimal liquid medium containing 0.1% casamino acids and 1 µg uracil ml^−1^ supplemented with and without 10 mM *myo*-inositol. The bacterial cells were harvested and disrupted to extract and purify the total RNA using RNeasy® Mini kit (Qiagen, Venlo, Netherlands). The total RNA was subjected to quantitative RT-PCR using RT-PCR Quick Master Mix (Toyobo, Osaka, Japan) and THUNDERBIRD® SYBR® qPCR Mix (Toyobo) as instructed by the provider to quantify the transcripts of the two tandem *iol* operons. The latter and former operons contain *gk1894* and *gk1899* as their respective first open reading frames, and the transcripts were amplified with specific primer pairs, gk1894RT-F/gk1894RT-R for *gk1894* and gk1899RT-F/gk1899RT-R for *gk1899* (Additional file 1). The quantified data was normalized by the comparative threshold cycle (C_t_) method using constitutive *gk0103* transcript as internal control [see Additional file 2], which encodes translation elongation factor G, amplified with the primer pair gk0103RT-F/gk0103RT-R (Additional file 1).

## Supplementary Information

**Additional file 1. **Primers used in this study.

**Additional file 2. **Standard curves showing quantity of total RNA vs. threshold cycle (Ct) for *gk0103* transcript. The total RNA samples were prepared from strains MK244 and MR01 cells grown in the presence (MI +) and absence (MI –) of *myo*-inositol, and subjected to quantitative RT-PCR analysis to detect the *gk0103* transcript. The four standard curves made for the samples are very similar to each other, indicating that the copy number of *gk0103* transcript is constant and reliable as the internal control.

## Data Availability

The datasets used and analyzed during the current study are available from the corresponding author on reasonable request.

## Abbreviations

CFU: Colony formation unit
LB: Lysogeny broth
C_t_: Threshold cycle
