# Supplementary material for: A novel method for transforming Geobacillus kaustophilus with a chromosomal segment of Bacillus subtilis transferred via pLS20-dependent conjugation
Source: Microb Cell Fact. 2022 Mar 8;21:34. doi: 10.1186/s12934-022-01759-8 (PMC8903633; doi:10.1186/s12934-022-01759-8)
Supplement: Supplementary file 1 — Additional file 1. Primers used in this study. [file 12934_2022_1759_MOESM1_ESM.docx]

**Additional file 1. Primers used in this study.**

| Primer name | Sequences (5′ to 3′) |
| --- | --- |
| aprE-D-f2 | gacagaggaattagatacattcgcgttaatcaacgtacaagcagctgcac |
| aprE-D-r2-nested | tgctttcgctgattacaacattggtgacgctgcct |
| aprE-U-f3-nested | caccgagctcatagcttgtcgcgatcacctcatcc |
| aprE-U-f1 | ccggtacttgccaccacatcataac |
| aprE-U-r | cagtaacctcatcaagccaagctacctctcgctatttccgtagagactcg |
| degA-U-f | gtagcttggcttgatgaggttactg |
| degA-U-r2 | catcggtcataaaatccgtatccttggttactttcatcgctcatcattc |
| degA-D-f2 | ctgcaaggcgattaagttgggtaacagtgaaatcgtaaggatgtgagcag |
| degA-D-r | cgcgaatgtatctaattcctctgtc |
| kan-f | aaggatacggattttatgaccgatg |
| kan-r2 | gttacccaacttaatcgccttgcag |
| kan-probe-f | taatacgactcactatagggtatggctctcttggtcgtc |
| kan-probe-r | tctgattccacctgagatgc |
| gk1894RT-F | ttttcgtcaacccttgaacc |
| gk1894RT-R | gagggatggcaagtacatcg |
| gk1899RT-F | attcctcgaggcttttgacc |
| gk1899RT-R | tgactcgggtgaaagtaggg |
| gk0103RT-F | catgactgacccgtatgtcg |
