# Supplementary material for: A novel method for transforming Geobacillus kaustophilus with a chromosomal segment of Bacillus subtilis transferred via pLS20-dependent conjugation
Source: Microb Cell Fact. 2022 Mar 8;21:34. doi: 10.1186/s12934-022-01759-8 (PMC8903633; doi:10.1186/s12934-022-01759-8)
Supplement: Supplementary file 2 — Additional file 2. Standard curves showing quantity of total RNA vs. threshold cycle (Ct) for gk0103 transcript. The total RNA samples were prepared from strains MK244 and MR01 cells grown in the presence (MI +) and absence (MI –) of myo-inositol, and subjected to quantitative RT-PCR analysis to detect the gk0103 transcript. The four standard curves made for the samples are very similar to each other, indicating that the copy number of gk0103 transcript is constant and reliable as the internal control. [file 12934_2022_1759_MOESM2_ESM.docx]

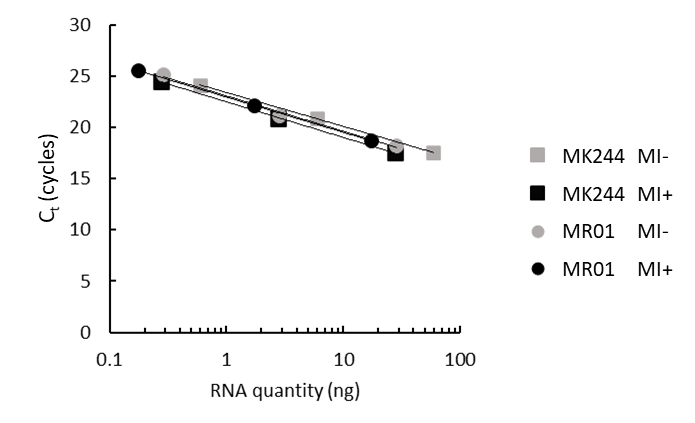


**Additional file 2.** Standard curves showing quantity of total RNA vs. threshold cycle (Ct) for *gk0103* transcript. The total RNA samples were prepared from strains MK244 and MR01 cells grown in the presence (MI +) and absence (MI –) of *myo*-inositol, and subjected to quantitative RT-PCR analysis to detect the *gk0103* transcript. The four standard curves made for the samples are very similar to each other, indicating that the copy number of *gk0103* transcript is constant and reliable as the internal control.
